# One‐year outcomes of rate versus rhythm control of atrial fibrillation in the Kerala‐AF Registry

**DOI:** 10.1002/joa3.13059

**Published:** 2024-05-21

**Authors:** Peter Calvert, Yang Chen, Ying Gue, Dhiraj Gupta, Jinbert Lordson Azariah, A. George Koshy, Geevar Zachariah, Gregory Y. H. Lip, Bahuleyan Charantharayil Gopalan, the Kerala AF Registry Investigators, Narayanan Namboodiri, A. Jabir, A. George Koshy, Geevar Zachariah, M. Shifas Babu, K. Venugopal, Eapen Punnose, K. U. Natarajan, Johny Joseph, C. Ashokan Nambiar, P. B. Jayagopal, P. P. Mohanan, Raju George, Govindan Unni, C. G. Sajeev, N. Syam, Anil Roby, Rachel Daniel, V. V. Krishnakumar, Anand M. Pillai, Stigi Joseph, G. K. Mini, Shaffi Fazaludeen Koya, Koshy Eapen, Raghu Ram, Cibu Mathew, Ali Faizal, Biju Issac, Sujay Renga, Jaideep Menon, D. Harikrishna, K. Suresh, Tiny Nair, S. S. Susanth, R. Anil Kumar, T. P. Abilash, P. Sreekala, E. Rajeev, Arun Raj, Ramdas Naik, Rajalekshmi, Anoop Gopinath, R. Binu, Jossy Chacko, P. T. Iqbal, N. M. Sudhir, Madhu Sreedharan, N. Balakrishnan, Muhammed Musthaffa, B. Jayakumar, Sheeba George, Anand Kumar, Thomas Mathew, V. K. Pramod, Muhammed Shaloob, Madhu Paulose Chandy, K. R. Vinod, Z. Sajan Ahamad, Pramod Mathew

**Affiliations:** ^1^ Liverpool Centre for Cardiovascular Science at University of Liverpool Liverpool John Moores University and Liverpool Heart and Chest Hospital Liverpool UK; ^2^ Department of Clinical Research Ananthapuri Hospitals and Research Institute Thiruvananthapuram India; ^3^ Department of Research Global Institute of Public Health Trivandrum India; ^4^ Cosmopolitan Hospital Trivandrum Kerala India; ^5^ Mother Hospital Thrissur Kerala India; ^6^ Danish Centre for Health Services Research, Department of Clinical Medicine Aalborg University Aalborg Denmark; ^7^ Department of Cardiology Ananthapuri Hospitals and Research Institute Thiruvananthapuram India

**Keywords:** atrial fibrillation, rate control, rhythm control, South Asia

## Abstract

**Background:**

There is ongoing debate around rate versus rhythm control strategies for managing atrial fibrillation (AF), however, much of the data comes from Western cohorts. Kerala‐AF represents the largest prospective AF cohort study from the Indian subcontinent.

**Objectives:**

To compare 12‐month outcomes between rate and rhythm control strategies.

**Methods:**

Patients aged ≥18 years with non‐transient AF were recruited from 53 hospitals across Kerala. Patients were stratified by rate or rhythm control. The primary outcome was a composite of all‐cause mortality, arterial thromboembolism, acute coronary syndrome or hospitalization due to heart failure or arrhythmia at 12 months. Secondary outcomes included bleeding events and individual components of the primary. Predictors of the composite outcome were analysed by logistic regression.

**Results:**

A total of 2901 patients (mean age 64.6 years, 51% female) were included (2464 rate control, 437 rhythm control). Rates of the primary composite outcome did not differ between groups (29.7% vs 30.0%; *p* = .955), nor did any component of the primary. Bleeding outcomes were also similar (1.6% vs 1.9%; *p* = .848). Independent predictors of the primary composite outcome were older age (aOR 1.01; *p* = .013), BMI <18 (aOR 1.51; *p* = .025), permanent AF (aOR 0.78; *p* = .010), HFpEF (aOR 1.40; *p* = .023), HFrEF (aOR 1.39; *p* = .004), chronic kidney disease (aOR 1.36; *p* < .001), and prior thromboembolism (aOR 1.31; *p* = .014).

**Conclusion:**

In the Kerala‐AF registry, 12‐month outcomes did not differ between rate and rhythm control cohorts.

## Graphical abstract

No significant difference was found between rate and rhythm control approaches to atrial fibrillation management in the 1‐year follow‐up of the Kerala‐AF cohort. Independent predictors of adverse outcomes included age, underweight BMI, heart failure, and chronic renal failure.

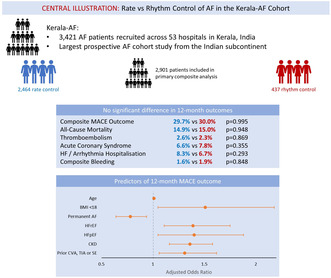

## 1 INTRODUCTION

Despite atrial fibrillation (AF) being very common in clinical practice globally, most study data come from Western cohorts. The Indian state of Kerala has seen rapid development and epidemiological transition recently, resulting in improved longevity and consequently a shift in mortality risk from communicable to non‐communicable causes such as cardiovascular disease.^1^, ^2^ The estimated mortality rate due to cardiovascular disease in Kerala is now higher than in many Western countries,^1^ emphasizing the need for effective treatment and prevention.

The Kerala‐AF registry^3^ was designed to provide a prospective analysis of patterns, treatment, and outcomes of AF in the Kerala region, with which to inform changes in practice. This is the largest prospective AF cohort from the Indian subcontinent.

International guidelines suggest AF should be managed with a holistic or integrated care approach, utilizing the Atrial Fibrillation Better Care (ABC) pathway.^4^, ^5^, ^6^ The ‘B’ component of the pathway involves a decision on whether to pursue restoration of sinus rhythm (rhythm control) or accept a state of permanent AF and focus on normalizing the heart rate (rate control).

In this ancillary analysis from the Kerala‐AF registry, we assessed 12‐month outcomes, as stratified by rate versus rhythm control strategies.

## 2 METHODS

### 2.1 Study design and cohort

The design of the Kerala‐AF registry has been described previously.^3^ Briefly, patients aged ≥18 years with documented AF were recruited between April 2016 and April 2017 across 53 hospitals in the Kerala region of India. Patients were recruited during attendance or admission to a recruiting hospital. Patients with transient AF (e.g., due to myocardial infarction, sepsis, or post‐operative AF) were excluded, as were critically ill patients with life expectancy <30 days.

The study was conducted in accordance with the Indian Council of Medical Research guidelines, and the ethical principles of the Declaration of Helsinki. All participants provided informed consent and only de‐identified data were shared for analysis.

### 2.2 Definition of rate versus rhythm control

Sites were asked to report rate or rhythm control on the study proforma, however discrepancies were noted during data analysis. Hence, for the purpose of this analysis, rhythm control was defined as (a) use of class I antiarrhythmic drugs; (b) use of catheter ablation; or (c) site definitively reported a rhythm control strategy. Thus, 117 patients classified as rhythm control were classified as having ‘permanent AF’. As the definition of permanent AF means that rhythm control has been discontinued, these patients were reclassified to ‘persistent AF’.

### 2.3 Study endpoints

The primary outcome in this analysis was a composite of major adverse cardiac events (MACE)—defined as all‐cause mortality, cerebrovascular accident (CVA), transient ischaemic attack (TIA), systemic embolism (SE), acute coronary syndrome (ACS) or hospitalization due to heart failure or arrhythmia—over 12 months of follow‐up.

Secondary outcomes included a composite of bleeding events, including gastrointestinal (GI) bleeds, intracranial haemorrhage (ICH), or other minor bleeding events. Individual endpoints within the primary composite outcome were also reported as secondary outcomes.

### 2.4 Statistical analysis

Continuous variables were described as mean ± standard deviation and compared using *t*‐tests. As all continuous variables were reasonably normally distributed, medians and non‐parametric tests were not applied. Categorical data were described using counts and/or percentages and compared using two‐sample proportion tests, Chi square tests or Fisher's exact test.

A loss to follow‐up was considerable in this dataset, we applied an algorithm to determine which patients to include in each outcome analysis, as follows: (a) if the patient had the event of interest, regardless of follow‐up duration, they were included; (b) if the patient completed 12‐month follow‐up without having the event, they were included; (c) if the patient died during follow‐up, they were included; (d) otherwise they were excluded. This approach ensured that patients with a known outcome, despite loss to follow‐up, could be included in analysis. Sensitivity analyses to assess the impact of loss to follow‐up were performed to ensure robustness of findings; (a) assuming all lost‐to‐follow‐up patients remained event‐free, and (b) assuming all lost‐to‐follow‐up patients experienced the primary composite endpoint.

Missing data were handled either by removal of the variable, simple imputation, or multivariable imputation by chained equations (MICE). As hospital ID was missing for 5 patients, these were classified into a separate ‘unknown hospital’ category. Patients with apparent considerable data entry errors were excluded.

The primary outcome was analyzed by logistic regression. AF management strategy, along with variables with *p* < .1 on univariable regression were entered into a multivariable logistic regression model. To account for clustering by hospital, a marginal regression model using generalized estimating equations with an exchangeable covariance structure was applied.

*p*‐Values <.05 were considered statistically significant. Statistical analysis was performed in Python and R.

## 3 RESULTS

The original Kerala‐AF dataset included 3421 patients. One record was removed due to data entry errors. Using the algorithm described above to account for loss to follow‐up, the primary outcome cohort consisted of 2901 patients (mean age 64.6 years; 51% female), of whom 2464 were assigned to a rate control strategy and 437 were assigned to a rhythm control strategy. The cohort sizes for secondary outcomes differed slightly due to the application of the loss to follow‐up algorithm for these different outcome measures.

Demographic and clinical differences between the primary outcome cohorts are shown in Tables 1 and 2. Hypertension was more common in the rhythm control group. Left atrial size was larger in the rate control group, as was history of thromboembolism, and valvular AF. Reasons for index consultation were similar in most cases, though the rate control group was more often related to valvular heart disease (7.8% vs 3.4%; *p* = .002). Most anticoagulation in both groups was achieved using Vitamin K anticoagulants (VKAs) such as Warfarin. Non‐vitamin K antagonist oral anticoagulants (NOACs) were infrequently used in either arm. Overall the cohort was high at risk for adverse events, with a high prevalence of chronic kidney disease and prior thromboembolism.

**TABLE 1:** Demographic differences between rate and rhythm cohorts.

	Rate (*n* = 2464)	Rhythm (*n* = 437)	*p*
Age (mean ± SD)	64.7 ± 13.0	64.4 ± 13.3	.688
Female sex (%)	52.4	49.9	.368
BMI (mean ± SD)	24.3 ± 3.8	24.5 ± 4.0	.341
Smoking status (%)			.619
Current	2.6	3.4	
Past	19.5	18.8	
Never	77.8	77.8	
Alcohol use (%)			.500
Current	13.7	15.6	
Past	4.5	5.0	
Never	81.8	79.4	
Heart failure (%)			.132
HFpEF	11.6	9.6	
HFrEF (LVEF <50%)	15.2	12.6	
None	73.2	77.8	
Co‐morbidities (%)			
Hypertension	52.8	60.4	**.004**
Diabetes mellitus	34.7	33.0	.503
Dyslipidaemia	42.9	44.4	.586
Ischaemic heart disease	35.8	37.1	.659
Chronic respiratory disease	21.4	18.8	.239
Chronic kidney disease	48.3	44.9	.202
Chronic liver disease	1.9	0.7	.099
Prior CVA, TIA or SE	15.4	11.0	**.020**
Prior bleeding event	7.6	6.4	.425
Symptomatic AF (%)	86.0	85.1	.683
CHA_2_DS_2_VASc score (mean ± SD)	2.8 ± 1.6	2.8 ± 1.6	.795
HASBLED score (mean ± SD)	2.3 ± 1.4	2.3 ± 1.4	.641

*Note*: Cohort differences are described for the primary outcome cohort only—see Sections 2 and 3 for details.

Abbreviations: CVA, cerebrovascular accident; HFpEF, heart failure with preserved ejection fraction; HFrEF, heart failure with reduced ejection fraction; SD, standard deviation; SE, systemic embolism; TIA, transient ischaemic attack.

**TABLE 2:** Clinical differences between rate and rhythm groups.

	Rate (*n* = 2464)	Rhythm (*n* = 437)	*p*
Valvular AF (%)	28.0	19.5	**<.001**
NYHA class III–IV (%)	18.2	16.2	.355
AF classification (%)			**<0.001**
Paroxysmal	34.4	63.8	
Persistent	13.8	36.2	
Permanent	51.8	‐	
Medications (%)			
Beta blocker	54.3	63.2	**.001**
Rate‐limiting CCB	23.3	4.1	**<.001**
Digoxin	37.8	16.0	**<.001**
Class I AAD	‐	9.6	**<.001**
Class III AAD	15.8	37.8	**<.001**
Vitamin K anticoagulant	65.9	56.5	**<.001**
Non‐VKA oral anticoagulant	6.1	5.3	.553
Antiplatelet	42.9	45.3	.368
Catheter ablation (%)	‐	2.5	**<.001**
Pacemaker implant (%)	4.7	4.1	.651
LA diameter (mm) (mean ± SD)	42.3 ± 7.7	40.3 ± 6.9	**<.001**
LV ejection fraction (mean ± SD)	56.3 ± 11.3	57.0 ± 11.1	.208
Reason for index consultation (%)			
AF	70.7	71.2	.901
Coronary disease	7.0	8.5	.314
Heart failure	4.6	5.3	.649
Hypertension	1.2	1.4	.971
Other/Not known	7.2	8.7	.327
Stroke/TIA/SE	1.4	1.6	.940
Valvular disease	7.8	3.4	**.002**

*Note*: Cohort differences are described for the primary outcome cohort only—see Sections 2 and 3 for details.

Abbreviations: AAD, anti‐arrhythmic drug; AF, atrial fibrillation; CCB, calcium channel blocker; LA, left atrial; LV, left ventricular; SD, standard deviation; SE, systemic embolism; TIA, transient ischaemic attack.

### 3.1 Outcomes

The primary composite MACE outcome did not differ significantly between the two groups (rate control 29.7% vs rhythm control 30.0%; *p* = .955). Similarly, there were no differences in the individual endpoints of thromboembolism (2.6% vs 2.3%; *p* = .869), acute coronary syndrome (6.6% vs 7.8%; *p* = .355) or hospitalization for heart failure or arrhythmia (8.3% vs 6.7%; *p* = .293).

There was also no difference in all‐cause mortality (rate control 14.9% vs rhythm control 15.0%; *p* = .948). Causes of death were mostly classified as cardiac in both groups (74.2% vs 78.5%; *p* = .539). Stroke accounted for 14.7% of deaths in the rate control group and 7.7% in the rhythm control group (*p* = .169). The remainder were classified as ‘other’ or ‘unknown’. The composite bleeding outcome was also no different between groups (rate control 1.6% vs rhythm control 1.9%; *p* = .848).

### 3.2 Predictors of the composite MACE outcome

The results of univariable and multivariable logistic regression analyses, predicting the composite MACE outcome, are shown in Table 3.

**TABLE 3:** Logistic regression models for the primary composite MACE outcome.

Parameter	Univariable odds ratio (95% CI)	*p*‐value	Multivariable adjusted OR (95% CI)	*p*‐value
Rhythm control strategy	0.98 (0.79–1.21)	.841	0.86 (0.67–1.10)	.238
Age (per year)	1.02 (1.01–1.03)	**<.001**	1.01 (1.00–1.02)	**.013**
Female sex	0.95 (0.82–1.10)	.475		
Weight category				
Normal (BMI 18–24)	Ref	Ref	Ref	Ref
Underweight (BMI <18)	1.56 (1.10–2.22)	**.013**	1.51 (1.05–2.18)	**.025**
Overweight (BMI 25–30)	0.87 (0.73–1.03)	.105	0.90 (0.76–1.08)	.271
Obese (BMI 31–40)	0.85 (0.61–1.17)	.312	0.94 (0.68–1.29)	.694
Morbidly Obese (BMI ≥40)	1.39 (0.53–3.68)	.503	1.60 (0.56–4.56)	.378
Smoking status				
Current	Ref	Ref		
Past	0.82 (0.52–1.27)	.369		
Never	0.88 (0.54–1.41)	.589		
Alcohol use				
Current	Ref	Ref		
Past	0.92 (0.59–1.45)	.735		
Never	1.11 (0.86–1.42)	.425		
AF classification				
Paroxysmal	Ref	Ref	Ref	Ref
Persistent	1.10 (0.85–1.42)	.460	1.13 (0.86–1.48)	.378
Permanent	0.79 (0.65–0.97)	**.021**	0.78 (0.64–0.94)	**.010**
Heart failure				
None	Ref	Ref	Ref	Ref
HFrEF	1.58 (1.25–2.00)	**<.001**	1.39 (1.11–1.75)	**.004**
HFpEF	1.47 (1.09–1.98)	**.011**	1.40 (1.05–1.88)	**.023**
Co‐morbidities				
Hypertension	1.12 (0.94–1.34)	.196		
Diabetes	1.27 (1.06–1.53)	**.011**	1.16 (0.96–1.41)	.132
Ischaemic heart disease	1.37 (1.20–1.57)	**<.001**	1.12 (0.96–1.30)	.161
Chronic kidney disease	1.66 (1.41–1.96)	**<.001**	1.36 (1.16–1.58)	**<.001**
Prior CVA, TIA or SE	1.28 (1.04–1.59)	**.023**	1.31 (1.06–1.62)	**.014**
Valvular AF	0.86 (0.65–1.13)	.279		
Medications				
Beta‐blocker	0.94 (0.81–1.11)	.478		
Rate‐limiting CCB	1.03 (0.84–1.26)	.766		
Digoxin	1.09 (0.90–1.33)	.379		
Class I AAD	1.03 (0.89–1.81)	.913		
Class III AAD	1.29 (1.11–1.50)	**.001**	1.16 (0.99–1.36)	.066
Vitamin K anticoagulant	0.83 (0.65–1.07)	.148		
Non‐VKA oral anticoagulant	0.79 (0.50–1.23)	.293		
Antiplatelet	1.07 (0.89–1.29)	.449		
Catheter ablation	0.50 (0.17–1.48)	.210		
Pacemaker implant	0.92 (0.60–1.42)	.710		
LA diameter (per mm)	1.00 (0.99–1.01)	.855		

Abbreviations: AAD, anti‐arrhythmic drug; AF, atrial fibrillation; BMI, body mass index; CCB, calcium channel blocker; CI, confidence interval; HFrEF, heart failure with reduced ejection fraction; HFpEF, heart failure with preserved ejection fraction; LA, left atrial; OR, odds ratio; VKA, vitamin K anticoagulant.

On *univariable regression*, age (*p* < .001), heart failure, both HFpEF and HFrEF (*p* < .001), diabetes mellitus (*p* = .011), ischaemic heart disease (*p* < .001), chronic kidney disease (*p* < .001), prior thromboembolism (*p* = .023) and use of class III antiarrhythmics (*p* = .001) were associated with increased risk of the composite MACE outcome. BMI was analyzed categorically as the linearity assumption was not met. As compared to a reference of normal BMI (18–24), being underweight significantly increased the risk of composite MACE events (*p* = .013). The other weight categories showed no significant association. Rhythm control strategy, as compared to rate control, was non‐significant (OR 0.98; 95% CI 0.79–1.21; *p* = .841).

Following multivariable adjustment, *independent predictors of the composite MACE outcome* were age (aOR 1.01; 95% CI 1.00–1.02; *p* = .013), BMI <18 (aOR 1.51; 95% CI 1.05–2.18; *p* = .025), permanent AF (aOR 0.78; 95% CI 0.64–0.94; *p* = .010), HFpEF (aOR 1.40; 95% CI 1.05–1.88; *p* = .023), HFrEF (aOR 1.39; 95% CI 1.11–1.75; *p* = .004), CKD (aOR 1.36; 95% CI 1.16–1.58; *p* < .001), and prior thromboembolism (aOR 1.31; 95% CI 1.06–1.62; *p* = .014).

### 3.3 Sensitivity analyses

Sensitivity analyses, shown in the supplementary material, demonstrated minor changes to statistical significance, but did not substantially alter our overall findings. In the minimal loss effect model (assuming all patients lost‐to‐follow‐up remained event‐free), the sole difference was that permanent AF was no longer significantly associated with the composite MACE outcome, however, the trend remained in the same direction. In the maximal loss effect model (assuming all patients lost‐to‐follow‐up experienced a composite MACE outcome), underweight BMI became non‐significant and overweight BMI became significant, and prior thromboembolism became non‐significant, though the overall point estimates and confidence intervals were broadly similar to the main analysis. Additionally, the point estimate of catheter ablation was much closer to no effect.

## 4 DISCUSSION

The main findings from this ancillary analysis from the largest prospective AF cohort from the Indian subcontinent, are as follows: (i) There was no difference in the primary composite MACE outcome, nor any specified secondary outcome, when stratified by rate versus rhythm control strategies; and (ii) Significant independent predictors of the composite MACE outcome on multivariable logistic regression analysis were older age, underweight BMI (<18), heart failure (both HFpEF and HFrEF), CKD and history of CVA, TIA or systemic thromboembolism. Permanent AF was inversely associated, whilst overweight/obese BMI categories trended towards the inverse association. Morbid obesity trended toward an increased risk but did not meet statistical significance.

Co‐morbidity burden, reflected in the CHA_2_DS_2_VASc score, is known to be associated with increased cardiovascular risk even in the absence of AF.^7^, ^8^ Prior thromboembolism (CVA, TIA or systemic embolism) is a well established highly significant risk factor, hence it represents 2 points in the CHA_2_DS_2_VASc score. Similarly, it is well known that heart failure and CKD portend a poor prognosis, hence these associations are logical. We discuss the other factors in more detail in the following sections.

Notably, the overall MACE and mortality rates in our study were very high; more than might be expected for this region. This is likely, in part, reflective of the fact that patients were recruited at the time of hospital admission, resulting in an overall ‘sicker’ cohort. This is demonstrated by the high rates of comorbidities such as hypertension, diabetes mellitus and chronic renal disease, as shown in Table 1.

### 4.1 The impact of rhythm control

There is increasing interest in the prognostic benefits of early AF rhythm control.^9^, ^10^, ^11^ Nonetheless, older studies failed to demonstrate any benefit to rhythm control over a rate control strategy.^12^ This may be due to evolution in the landscape of preventative medicine, as well as improvements and more widespread use of techniques such as catheter ablation.

Ablation is known to be more effective than antiarrhythmic drugs at maintaining sinus rhythm and, once the small up‐front risk is past, there is no long‐term exposure to potentially toxic drug side‐effects. Rates of catheter ablation were considerably lower in Kerala‐AF than those seen in contemporary Western cohorts—just 15 patients (2.7%) underwent this procedure, which is not dissimilar to the 5.2% seen in the negative AFFIRM study.^12^ This contrasts with almost 20% by 2‐year follow‐up in the EAST AFNET‐4 study, which showed a reduction in the primary composite endpoint of cardiovascular death, stroke, or hospitalization for heart failure or acute coronary syndrome (HR 0.79; 95% CI 0.66–0.94; *p* = .005).^9^ The point estimate for catheter ablation in our regression model (OR 0.50) suggests potential benefit, however the confidence intervals are wide due to low numbers. This may suggest that improving access to ablation could contribute to better outcomes in rhythm control, though this would ideally need to be demonstrated in prospective randomized studies.

Interestingly, permanent AF (vs paroxysmal) was associated with reduced odds of the composite MACE outcome (aOR 0.78 [95% CI 0.64–0.94]; *p* = .010), whilst the use of class III antiarrhythmics, which were more frequently used in the rhythm control arm (37.8% vs 15.8%; *p* < .001) was associated with increased odds (aOR 1.16 [95% CI 0.99–1.36]; *p* = .066), though statistical significance was borderline. It is possible that the prognostic benefits of rhythm control were, in part, offset by the toxic effects of drugs such as Amiodarone, which are well described. This may also reflect unmeasured confounding in the data, with overall healthier patients left on a rate control strategy. Indeed, permanent AF patients were younger (mean age 62.8 vs paroxysmal 67.0) and had lower rates of some co‐morbidities such as hypertension (37% vs 45%), diabetes (39.5% vs 44%) and IHD (35.6% vs 46.6%). The reasons for this are unclear; it may be speculated that this could reflect a symptom‐based approach to AF management—that is, asymptomatic patients may not be referred for rhythm control—however, reported symptoms were somewhat more common in the permanent group (42% vs 39.8%) and were no different between the rate and rhythm control cohorts (86% vs 85%; *p* = .683). As the very long‐term effects of AF, particularly on neurocognitive outcomes, are increasingly recognized,^13^ these figures may reflect a need to promote a rhythm control strategy in the younger and healthier populations.

Furthermore, comprehensive management following the Atrial fibrillation Better Care (ABC) pathway, especially in terms of cardiovascular risk optimization—cannot be overlooked. The findings in the Kerala‐AF registry may relate to relative undertreatment. For example, NOACs were rarely utilized and many patients were not anticoagulated despite elevated stroke risk scores. Given the high rates of cardiovascular disease in the Kerala state, primary preventative efforts are essential. A recent survey showed that many preventative medications are either unaffordable or unavailable within Kerala,^14^ demonstrating the need for improvement.

Our analysis demonstrates that, at this point in time and over 1 year of follow‐up, rhythm control of AF does not confer a prognostic advantage in the Kerala‐AF cohort. The focus of healthcare improvements going forward should likely be on primary prevention to optimize overall cardiovascular risk. Improving access to modern therapies, such as NOACs and catheter ablation, may also be of benefit.

### 4.2 The effect of BMI on cardiovascular risk

In our study, underweight BMI was associated with an increased risk of composite MACE outcomes (aOR 1.51 [95% CI 1.05–2.18]; *p* = .025). This may be explained by malnourishment, which is known to be associated with increased cardiovascular risk.^15^, ^16^ Alternatively, patients with chronic health conditions, such as cancer, frequently lose weight rapidly and thus may fall into the underweight category, resulting in bias towards mortality risk.

Notably, the point estimates for overweight and obese BMIs were inversely associated with the primary composite outcome, but not to statistical significance. It is well established that obesity is a risk factor for cardiovascular disease. In the past, the concept of the “obesity paradox” has been described—that is, that obesity may somehow be protective in those with established cardiovascular disease. This paradox is partly due to the fact that BMI fails to account for body composition—for example, a bodybuilder may be classed as overweight due to high volume of lean muscle. A recent study showed that alternative measures of adiposity, such as waist‐to‐height ratio, may be more reliable.^17^

In general, promoting a target normal BMI (18–24) by eating a healthy diet and regularly exercising, remains the most logical recommendation.

## 5 LIMITATIONS

Whilst this study analyzed prospective registry data, it is subject to some limitations. In particular, there was no randomization to rate or rhythm control—treatment decisions were made by a physician on clinical grounds, and this cannot be adequately controlled through statistical adjustment alone. Similarly, the exact management strategy (rate or rhythm) applied to each individual was unclear in some cases; however, we feel that our algorithm, as described in our methods, should reasonably predict the relevant strategy. Whilst there are numerous prospective studies with randomized assignment to rate or rhythm control, these studies are primarily performed in first‐world Western cohorts, hence a strength of our study is an analysis of real‐world data from a South Asian cohort in a region undergoing epidemiological transition.

Kerala‐AF is currently the largest South Asian AF registry, however our findings may not be generalizable outside of Kerala, as the study was limited to this region. However, these findings may provide useful insight into the impact of AF therapies in other regions undergoing epidemiological transition.

The registry did not capture the duration of AF prior to enrolment, nor what strategies had been attempted in the past—for example, it is unclear how many rate control patients had failed attempted rhythm control, as opposed to those who were initially treated with rate control. We also did not have access to AF burden or monitoring data, nor time from diagnosis to rhythm control initiation, which precludes conclusions around the effects of ‘early rhythm control’, and the relative success of rhythm control cannot be reliably ascertained. Similarly, many patients in the rhythm control arm did not undergo catheter ablation and were not taking antiarrhythmic drugs—in most cases, this is likely due to them maintaining sinus rhythm without requiring antiarrhythmic therapies—though we cannot confirm this from the available data. Our findings are limited to 12‐month follow‐up, however, longer‐term follow‐up will be reported in the future.

## 6 CONCLUSION

In high‐risk AF patients from the Kerala region of India, rhythm control, as compared to rate control, did not significantly affect 12‐month outcomes. This may relate to the current epidemiological transition phase of the region, and our findings suggest that improving access to modern therapies—such as NOACs and catheter ablation—and primary prevention of cardiovascular disease in particular, should be the focus of healthcare development going forward.

Older age, underweight BMI, heart failure, chronic kidney disease, and history of thromboembolic disease were independently associated with major adverse cardiovascular outcomes. These factors should be considered when assessing individuals with greater comorbidity burden, as they may stand to gain the most benefit from aggressive prevention strategies.

## FUNDING INFORMATION

The Kerala‐AF registry was supported by the Kerala Chapter of Cardiological Society of India through a one‐time research grant No. CSI/IEC/2017. No funding was received towards the analysis and writing of this manuscript.

## CONFLICT OF INTEREST STATEMENT

GYHL reports: Consultant and speaker for BMS/Pfizer, Boehringer Ingelheim, Daiichi‐Sankyo, Anthos. No fees are received personally. GYHL is a National Institute for Health and Care Research (NIHR) Senior Investigator and co‐principal investigator of the AFFIRMO project on multimorbidity in AF, which has received funding from the European Union's Horizon 2020 research and innovation programme under grant agreement No 899871. DG reports: Speaker for Boehringer Ingelheim, Biosense Webster and Boston Scientific. Proctor for Abbott. Research Grants from Medtronic, Biosense Webster, and Boston Scientific. The other authors report no conflicts of interest.

## Supporting information


Table S1.
