# Supplementary material for: One‐year outcomes of rate versus rhythm control of atrial fibrillation in the Kerala‐AF Registry
Source: J Arrhythm. 2024 May 21;40(4):822–9. doi: 10.1002/joa3.13059 (PMC11317704; doi:10.1002/joa3.13059)
Supplement: Supplementary file 1 — Table S1. [file JOA3-40-822-s001.docx]

# Supplementary Material

**Supp. Table 1 – Sensitivity analyses for the primary composite outcome, assessing the impact of loss-to-follow-up (univariable odds ratio using GEE model as described in the manuscript)**

| Parameter | Main Study  OR (95% CI) | Minimal Loss Effect  OR (95% CI) | Maximal Loss Effect  OR (95% CI) |
| --- | --- | --- | --- |
| Rhythm Control Strategy | 0.98 (0.79-1.21) | 0.95 (0.78-1.15) | 1.06 (0.89-1.27) |
| Age (per year) | **1.02 (1.01-1.03)** | **1.02 (1.01-1.02)** | **1.01 (1.01-1.02)** |
| Female Sex | 0.95 (0.82-1.10) | 0.98 (0.86-1.13) | 0.89 (0.77-1.04) |
| Weight Category |  |  |  |
| Normal (BMI 18-24) | ref | ref | ref |
| Underweight (BMI <18) | **1.56 (1.10-2.22)** | **1.65 (1.16-2.34)** | 1.26 (0.91-1.74) |
| Overweight (BMI 25-30) | 0.87 (0.73-1.03) | 0.93 (0.78-1.10) | **0.80 (0.68-0.95)** |
| Obese (BMI 31-40) | 0.85 (0.61-1.17) | 0.89 (0.66-1.19) | 0.88 (0.65-1.18) |
| Morbidly Obese (BMI ≥40) | 1.39 (0.53-3.68) | 1.06 (0.34-3.24) | 1.82 (0.66-4.99) |
| Smoking Status |  |  |  |
| Current | ref | ref | ref |
| Past | 0.82 (0.52-1.27) | 0.87 (0.53-1.42) | 0.79 (0.60-1.07) |
| Never | 0.88 (0.54-1.41) | 0.92 (0.55-1.55) | 0.80 (0.58-1.11) |
| Alcohol Use |  |  |  |
| Current | ref | ref | ref |
| Past | 0.93 (0.59-1.45) | 0.89 (0.59-1.35) | 1.04 (0.71-1.52) |
| Never | 1.11 (0.86-1.42) | 1.09 (0.86-1.37) | 1.11 (0.90-1.37) |
| AF Classification |  |  |  |
| Paroxysmal | ref | ref | ref |
| Persistent | 1.10 (0.85-1.42) | 1.07 (0.83-1.38) | 1.16 (0.94-1.44) |
| Permanent | **0.79 (0.65-0.97)** | 0.83 (0.68-1.01) | **0.79 (0.66-0.95)** |
| Heart Failure |  |  |  |
| None | ref | ref | ref |
| HFrEF | **1.58 (1.25-2.00)** | **1.49 (1.17-1.89)** | **1.47 (1.20-1.79)** |
| HFpEF | **1.47 (1.09-1.98)** | **1.38 (1.05-1.82)** | **1.43 (1.08-1.89)** |
| Co-morbidities |  |  |  |
| Hypertension | 1.12 (0.94-1.34) | 1.13 (0.95-1.34) | 1.09 (0.94-1.26) |
| Diabetes | **1.27 (1.06-1.53)** | **1.24 (1.06-1.47)** | **1.21 (1.02-1.43)** |
| Ischaemic Heart Disease | **1.37 (1.20-1.57)** | **1.39 (1.21-1.60)** | **1.23 (1.05-1.43)** |
| Chronic Kidney Disease | **1.66 (1.41-1.96)** | **1.59 (1.34-1.89)** | **1.49 (1.32-1.69)** |
| Prior CVA, TIA or SE | **1.28 (1.04-1.59)** | **1.29 (1.05-1.59)** | 1.18 (0.97-1.45) |
| Valvular AF | 0.86 (0.65-1.13) | 0.88 (0.68-1.14) | 0.87 (0.69-1.11) |
| Medications |  |  |  |
| Beta Blocker | 0.94 (0.81-1.11) | 0.94 (0.81-1.09) | 0.98 (0.85-1.12) |
| Rate-limiting CCB | 1.03 (0.84-1.26) | 1.04 (0.85-1.26) | 1.00 (0.85-1.18) |
| Digoxin | 1.09 (0.90-1.33) | 1.08 (0.89-1.30) | 1.08 (0.91-1.28) |
| Class I AAD | 1.03 (0.89-1.81) | 1.18 (0.70-1.99) | 0.89 (0.51-1.55) |
| Class III AAD | **1.29 (1.11-1.50)** | **1.23 (1.07-1.43)** | **1.24 (1.05-1.47)** |
| Vitamin K Anticoagulant | 0.83 (0.65-1.07) | 0.86 (0.69-1.08) | 0.82 (0.66-1.03) |
| Non-VKA Anticoagulant | 0.79 (0.50-1.23) | 0.82 (0.55-1.24) | 0.83 (0.53-1.31) |
| Antiplatelet | 1.07 (0.89-1.29) | 1.09 (0.91-1.30) | 1.06 (0.91-1.23) |
| Catheter Ablation | 0.50 (0.17-1.48) | 0.42 (0.13-1.41) | 0.96 (0.43-2.16) |
| Pacemaker Implant | 0.92 (0.60-1.42) | 1.00 (0.66-1.52) | 0.81 (0.56-1.18) |
| LA Diameter (per mm) | 1.00 (0.99-1.01) | 1.00 (0.99-1.02) | 1.00 (0.98-1.01) |

The primary analysis was re-run for this table, including all patients lost-to-follow-up and assuming that they a) did not have the outcome (minimal loss effect), or b) did have the outcome (maximal loss effect). This determines the minimum and maximum impact of loss-to-follow-up respectively. Odds ratios are provided for each model, with significant p-values (<0.05) highlighted in bold.
